# South‐to‐Southeast Expansion of HIV‐1 Subtype C in Brazil

**DOI:** 10.1002/jmv.70580

**Published:** 2025-08-29

**Authors:** Daniel Polita, Laise de Moraes, Marta Giovanetti, Filipe Ferreira de Almeida Rego, Gabriel Carvalho, Luciane Amorim Santos, Ricardo Khouri, Dennis Maletich Junqueira

**Affiliations:** ^1^ Programa de Pós‐Graduação em Ciências Biológicas: Bioquímica Toxicológica (PPGBTox), Laboratório de Bioinformática e Evolução Viral Universidade Federal de Santa Maria (UFSM) Santa Maria Rio Grande do Sul Brazil; ^2^ Instituto Gonçalo Moniz, Fundação Oswaldo Cruz, Rua Waldemar Falcão Candeal Bahia Brazil; ^3^ Programa de Pós‐Graduação em Ciências da Saúde, Faculdade de Medicina da Bahia, Universidade Federal da Bahia, Praça Ramos de Queirós, s/n, Largo do Terreiro de Jesus Salvador Bahia Brazil; ^4^ Department of Sciences and Technologies for Sustainable Development and One Health Universita Campus Bio‐Medico di Roma Rome Lazio Italy; ^5^ Oswaldo Cruz Institute, Oswaldo Cruz Foundation (Fiocruz) Rio de Janeiro Brazil; ^6^ Escola Bahiana de Medicina e Saúde Pública, Avenida Dom João VI Brotas São Paulo Brazil; ^7^ Faculdade de Medicina, Universidade Federal da Bahia, Av. Reitor Miguel Calmon, S/N–Vale do Canela Salvador Bahia Brazil; ^8^ Rega Institute for Medical Research, Department of Immunology, Microbiology and Transplantation, KU Leuven Leuven Belgium

**Keywords:** Brazil, epidemic, HIV‐1, molecular phylogenetics, subtype C, transmission clusters

## Abstract

HIV‐1 group M subtype C (HIV‐1C) is the predominant genetic variant in southern Brazil but has been sparsely detected in other regions of the country. Our study aimed to identify HIV‐1C transmission links across Brazil using phylogenetic reconstruction and to infer trends in geographical patterns and dissemination beyond the southern region. We retrieved 3693 HIV‐1C (partial *pol*) sequences from NCBI and LANL databases and applied maximum likelihood phylogenetic reconstruction to infer transmission clusters, using clade confidence (SH‐aLRT) and intracluster genetic distance as critical parameters. Our results suggest that the Southern states of Brazil have played a key role in fueling the HIV‐1C epidemic in São Paulo, particularly through transmissions from Paraná and Santa Catarina. In contrast, despite having one of the highest HIV‐1C prevalence rates of Brazil, Rio Grande do Sul exhibited a highly concentrated epidemic with limited epidemiological linkages to other states. São Paulo seems to be a crucial hub for HIV‐1C dissemination, connecting the epidemic in the southern states with other regions. Central states may act as secondary hubs, facilitating connections between the southeastern and northeastern regions. This study enhances the understanding of the geographical dynamics and expansion patterns of HIV‐1C in Brazil, emphasizing the significance of regional epidemiological connections.

## 1 Introduction

A key characteristic of HIV is its remarkable ability to generate genetic diversity [1, 2]. The genetic spectrum of HIV consists of two main variants: HIV‐1 and HIV‐2. HIV‐1, responsible for most infections worldwide, is subdivided into four distinct groups (M, N, O, and P), which together comprise a wide range of subtypes, intersubtypes and recombinant forms [3, 4]. Group M, also known as the pandemic group, accounts for over 90% of HIV cases globally and includes 10 subtypes, designed as A to L (A, B, C, D, F, G, H, J, K, and L) [5]. Within this group, subtype C has stood out due to its rapid global expansion between 1990 and 2009, particularly in countries such as India, Ethiopia, and South Africa [6]. Although its prevalence slightly declined after 2010, subtype C still represents nearly half of global HIV‐1 infections [7].

In Brazil, the HIV‐1 epidemic is characterized by the presence of subtypes B, F1, and C, along with recombinant forms BC and BF [8]. Subtype B predominates across most of the country, except in the southern states of Rio Grande do Sul, Santa Catarina, and Paraná, where subtype C (HIV‐1C) is the most prevalent [9, 10]. The HIV‐1C is estimated to have been introduced into Brazil in the 1970s, likely originated from the eastern African country of Burundi and first appearing in Paraná [11, 12]. From the 1980s onwards, the spread from Paraná to the neighboring states of Santa Catarina and Rio Grande do Sul led HIV‐1C to rapidly become the dominant subtype in southern Brazil [13]. Historically, HIV‐1C has remained largely restricted to this region [14, 15], reaching a significant proportion of infections ‐ approximately 66.2% in Santa Catarina, 44.7% in Rio Grande do Sul, and 36.8% in Paraná [8]. However, sporadic cases have been reported nationwide in recent years [16, 17, 18, 19, 20, 21, 22], particularly in São Paulo, a key state in the Southeast. Epidemiological studies indicate a notable presence of HIV‐1C in São Paulo [23, 24], raising concerns about a potential localized emerging epidemic.

Southern Brazil, the epicenter of the country's HIV‐1C epidemic, is also characterized by elevated HIV detection rates that surpass the national average (Santa Catarina: 29.6 cases/100,000; Rio Grande do Sul: 26.0; Paraná: 21.3; national average: 17.8) [25]. However, the specific factors that facilitated the rapid and successful dissemination of HIV‐1C in this particular region remain an open question. Given the high degree of genetic mixture in the Brazilian population [26, 27, 28], it is unlikely that genetic differences between southern and other Brazilian populations alone explain the high prevalence of subtype C in the region.

Local epidemiological factors combined with social and behavioral characteristics likely played a fundamental role in the HIV‐1C dissemination throughout Southern Brazil. Key factors such as the historical association between subtype C and heterosexual transmission [9, 10, 14, 29, 30, 31], high female‐to‐male infection ratio (1.27:1) compared to other Brazilian regions and significant mother‐to‐child transmission [27] may have shaped the transmission patterns of HIV‐1C in southern Brazil. As suggested by Souto et al. (2021), the sociocultural and behavioral conditions in southern Brazil may have contributed to the spread of subtype C, similar to patterns observed in African and Asian regions where this subtype is also prevalent [27]. Additionally, evidence suggests that individuals infected with HIV‐1C have higher CD4^+^ T‐cell counts than those infected with other subtypes, such as B or F [18, 32], and experience slower disease progression. This prolonged asymptomatic period may increase transmission opportunities [33]. The interplay between local epidemiological and sociobehavioral factors, along with the molecular characteristics of HIV‐1C may at least partially explain its widespread dissemination in southern Brazil.

Monitoring the spread of HIV‐1C within and beyond southern Brazil, is crucial for public health, as it may have significant epidemiological implications. However, few studies have investigated HIV‐1C outside the southern region, resulting in a knowledge gap regarding its epidemiological dynamics in Brazil [14, 27, 30]. To address these gaps, we reconstructed the phylogenetic history of HIV‐1C in Brazil and inferred the circulating transmission chains, enabling us to identify transmission flows between states and patterns of viral population growth. These insights contribute to a better understanding of the epidemiological dynamics of HIV‐1C in Brazil.

## 2 Materials and Methods

### 2.1 Data Set Compilation

A total of 12 554 sequences were included in the initial data set, comprising all available HIV‐1C sequences isolated in Brazil and international sequences retrieved via BLAST search. This data set consisted of 3292 sequences from NCBI and 9262 sequences from the Los Alamos National Laboratory database. After applying strict exclusion criteria, removing sequences with ambiguities, duplicates, non‐subtype C variants, evidence of recombination, excessive shortness, and poor alignment, a final set of 3693 sequences were selected for phylogenetic analysis. Of these, 2782 sequences were generated from Brazil and 911 from other countries, primarily in Africa. Sequence alignment was performed using MAFFT v7.520 [34].

### 2.2 Phylogenetic Reconstruction

Phylogenetic reconstruction was conducted using the maximum likelihood (ML) method implemented in IQ‐TREE v1.6.12 [35]. To determine the optimal nucleotide substitution model, ModelFinder was used [36], resulting in the selection of the GTR + F + I + G4 model, which incorporates a general time reversible (GTR) substitution model, a proportion of invariable sites (I), and a gamma distribution (G4) with four rate categories to account for rate heterogeneity among sites [37]. Branch support values were assessed using the approximate likelihood‐ratio (aLRT) SH‐like test [38]. The final phylogeny was visualized, manipulated, and edited using FigTree v1.4.4.

### 2.3 Identification of Transmission Clusters

To explore HIV‐1C transmission dynamics in Brazil, transmission clusters were inferred from the ML phylogeny using ClusterPicker v1.2 [39]. Clusters were defined as monophyletic clades within the major Brazilian subtype C lineage, presenting an SH‐aLRT node support value ≥ 85 and an average intracluster genetic distance < 0.03 substitutions per site.

Given the arbitrary nature of clustering parameters [40], sensitivity analysis was performed by testing twelve different intracluster genetic distance values (0.005, 0.01, 0.015, 0.02, 0.025, 0.03, 0.035, 0.04, 0.045, 0.05, 0.055, 0.06) and four SH‐aLRT support values (80, 85, 90, and 95). Graphical analysis revealed that intracluster genetic distances > 0.03 led to increased variability in the number of identified clusters, independent of node support. Thus, using an intracluster genetic distance of 0.03, and a minimum values‐aLRT support of 85 yielded the most robust clustering results (Supporting information Figure 1). To understand the replicability of this parameter, we also used an intracluster genetic distance of 0.02 for comparison.

### 2.4 Bayesian Analysis

To estimate relative diversity and the effective population size of viral populations circulating in Brazil, Bayesian phylogenetic reconstruction was performed using BEAST v1.10.4 [41]. Four datasets, including all sequences from four different states (São Paulo, Paraná, Santa Catarina and Rio Grande do Sul), were analyzed individually under an uncorrelated relaxed molecular clock and a non‐parametric Bayesian Skygrid demographic model [42]. MCMC chains were run for 3.0 ×108 steps, with subsampling every 30 000 iterations. Parameter convergence was assessed using TRACER v1.7.2 [43] and the GMRF Skyride reconstruction plot was visualized using R v4.2.1.

### 2.5 Statistical Analysis of Temporal Trend of Transmission Cluster Growth

To evaluate the temporal trend of transmission cluster growth in the states of São Paulo, Paraná, Santa Catarina, and Rio Grande do Sul, Spearman correlation coefficients were calculated using the SciPy library in Python for both 2% and 3% genetic distance (GD) thresholds. Differences between the total number of sequences and the number of clustered sequences in each state, were assessed using a chi‐square test in R v4.2.1.

## 3 Results and Discussion

After its initial establishment in southern Brazil, HIV‐1C has historically shown limited dissemination to other regions [13, 14]. However, epidemiological reports of subtype C cases across different Brazilian states raise concerns about a possible shift in the national HIV landscape [16, 18, 19, 22, 23, 44]. In this study, we applied a phylogenetic approach to identify HIV‐1C transmission clusters in Brazil. Transmission clusters are defined as sets of sequences that are phylogenetically aggregated in a nonrandom manner, linked by epidemiological factors [45]. Given the lack of consensus on methods and parameters for inferring phylogenetic transmission clusters, which often rely on arbitrary thresholds, we sought to minimize subjectivity in our analysis. To achieve this, we tested different values for node support (SH‐aLRT) and genetic distance (GD). As the maximum genetic distance cut‐off is relaxed, more sequences are added to the clusters, since incorporating more sequences into each cluster and/or merging smaller clusters enables the identification of larger clusters [46]. Based on this result we judged the within‐cluster maximum genetic distance of 0.03 and SH‐aLRT ≥ 85 to be appropriate in detecting these clusters (Supporting information Figures 1 and 2).

**Figure 1:**
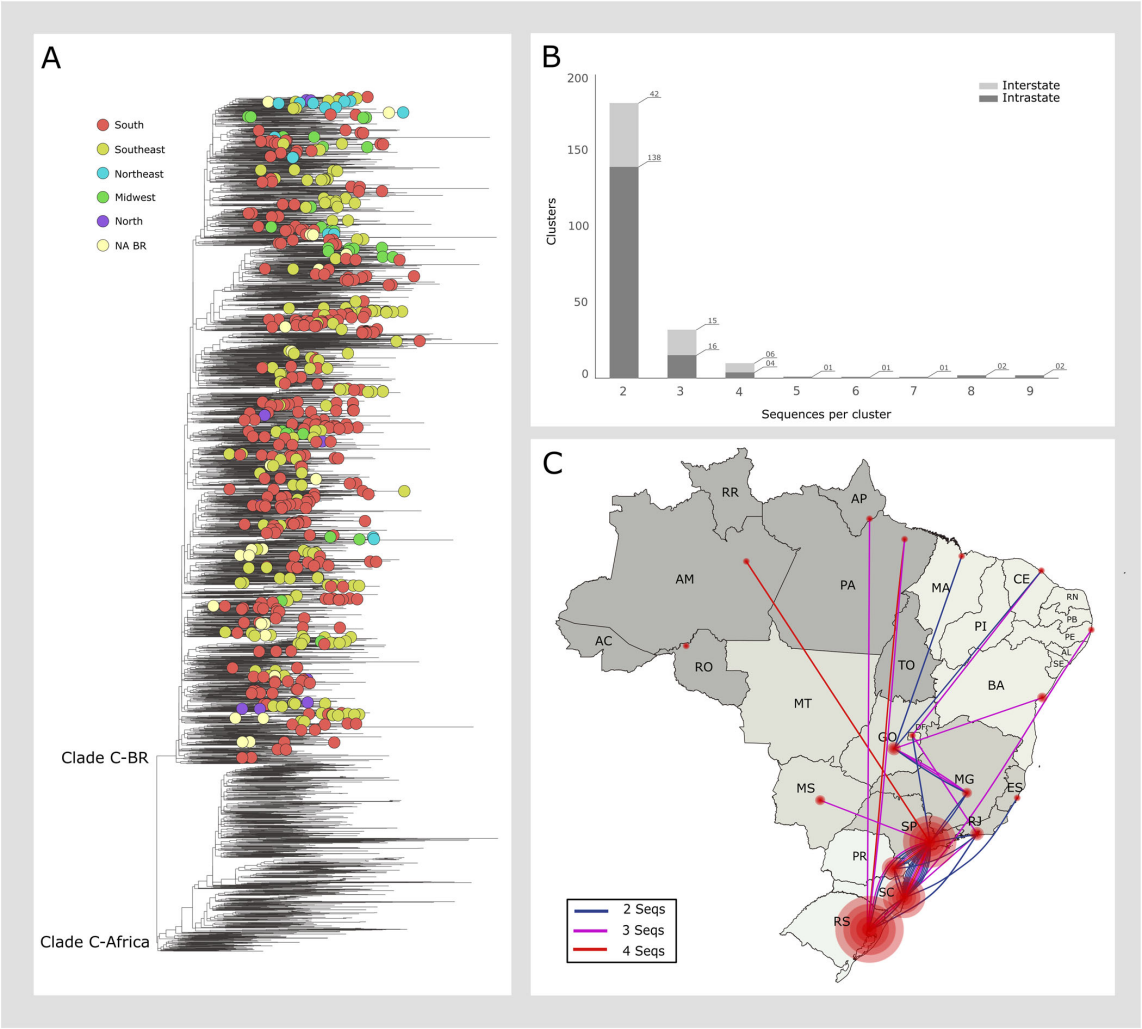
Quantitative and geographic distribution of transmission clusters identified in the HIV‐1 subtype C phylogenetic reconstruction encompassing sequences isolated in Africa and Brazil (1998–2017). (A) Maximum likelihood phylogenetic reconstruction of 3693 HIV‐1 subtype C pol sequences. Clustered sequences are highlighted and colored by region of sampling in Brazil (clustering criteria: node support ≥ 85 and maximum genetic distance = 0.03). (B) Number of clusters and total individuals included in Brazilian transmission clusters. Phylogenetic clusters are categorized as intrastate (dark gray) or interstate (light gray). (C) Geographic distribution of Brazilian transmission clusters. Size of the red circles are proportional to the amount of subtype C sequences in each state.

**Figure 2:**
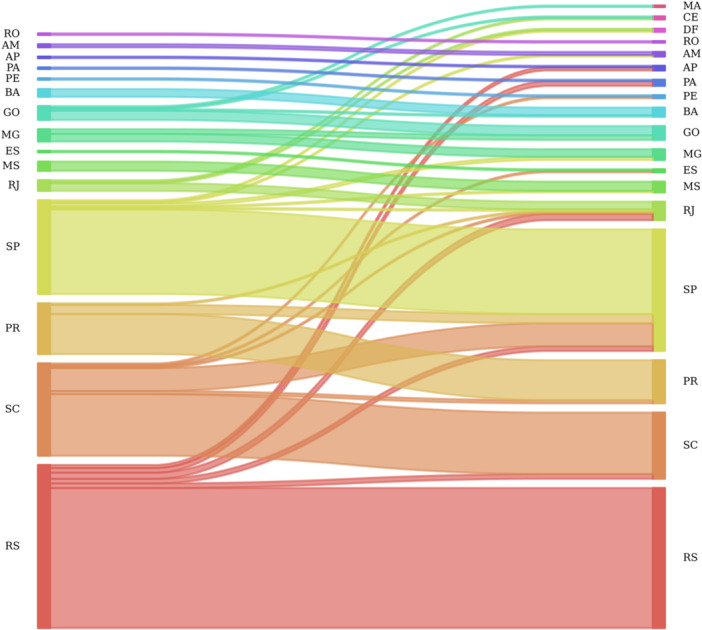
Sankey Diagram representing the spatial distribution of HIV‐1 subtype C sequences included in transmission clusters, Brazil. Brazilian states: AP (Amapá), AM (Amazonas), BA (Bahia), CE (Ceará), ES (Espírito Santo), GO (Goiás), MA (Maranhão), MS (Mato Grosso do Sul), MG (Minas Gerais), PA (Pará), PR (Paraná), PE (Pernambuco), RJ (Rio de Janeiro), RS (Rio Grande do Sul), RO (Rondônia), SC (Santa Catarina), SP (São Paulo).

**Figure 3:**
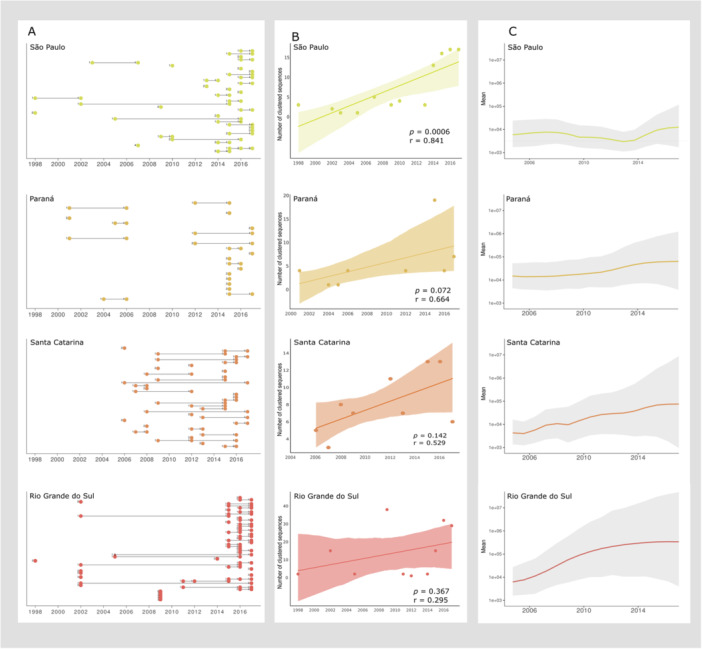
Temporal distribution of transmission clusters and viral growth of HIV‐1 subtype C in São Paulo, Paraná, Santa Catarina and Rio Grande do Sul. (A) Temporal distribution of intrastate transmission clusters. (B) Correlation between the number of sequences within transmission clusters and the year of sampling for transmission clusters (Intrastate cluster sequences only). (C) Effective population size of HIV‐1C for four states in Brazil (total number of sequences, clustered and non‐clustered).

We identified 228 Brazilian HIV‐1C clusters, comprising 545 sequences (Figure 1A,B, Supporting information Table 1). Most clusters (72.36%, 165 out of 228) were classified as intrastate, involving transmissions within the same state, while interstate clusters (transmission among different states) accounted for 27.6% (63 of 228, Figure 1B). The majority of HIV‐1C sequences in Brazilian transmission clusters (77.5%, 428 of 545 sequences) were isolated in the southern states (Rio Grande do Sul, Santa Catarina, and Paraná) or in São Paulo in the southeast (Figure 1C). Most identified clusters were intrastate (Figure 2), with sequences predominantly sampled after 2013 (Figure 3A), this pattern is particularly evident in Rio Grande do Sul.

In Rio Grande do Sul, historically one of the Brazilian states most affected by HIV‐1C, we identified 60 transmission clusters involving 155 sequences. Of these, 85% (51 out of 60) were intrastate, while only 15% (9 out of 60) involved sequences from other states. These findings support the hypothesis that HIV‐1C in Rio Grande do Sul represents a highly localized epidemic with limited impact on the national HIV landscape. As suggested by Delatorre et al. (2013), the epidemic in Rio Grande do Sul primarily results from the in‐situ dissemination of a single sub‐lineage that emerged around 1980 and has remained largely restricted to the state [11].

In Santa Catarina, we observed multiple transmission events to other states, mainly to São Paulo. A similar but less pronounced epidemiological pattern was observed in the state of Paraná. We hypothesize that São Paulo is a key state in the epidemiological landscape of HIV‐1C in Brazil. The epidemic of subtype C in the state of São Paulo establishes epidemiological connections with several states across Brazil, particularly with the southern states. However, the southern state of Rio Grande do Sul, which accounts for the higher proportion of sequences in our study (*n* = 844; 22.85%), stands out as an exception. The apparent isolation of the HIV‐1C epidemic in Rio Grande do Sul in relation to São Paulo could be at least partially explained by differences in the key populations driving subtype C transmission in each state (Figure 2). In Rio Grande do Sul, HIV‐1C is primarily linked to heterosexual transmission [14, 32], while in São Paulo the epidemic seems to be characterized by a recent expansion associated with young MSM networks [13]. While keeping in mind that the dissemination of HIV‐1C in different locations is multifactorial and includes biological, epidemiological, sociodemographic and cultural factors among others, it would be plausible to suggest the existence of social and behavioral barriers, such as described, that limit connectivity between the transmission networks of the two states.

Our results further corroborated the hypothesis that São Paulo functions not only as a recipient of viral influx from the Southern states, but also as a key transmission hub, facilitating HIV‐1C dissemination across Brazil [27]. We highlight the potential role played by Goiás, a state located outside the south‐southeast axis, in the dissemination of subtype C in Brazil. Eleven clusters were characterized in the Central West state, six of which were interstate, establishing epidemiological connections with the Southeastern state of Minas Gerais and the Northeastern states of Maranhão, Bahia and Ceará. These results suggest that Goiás may act as a secondary transmission hub, connecting the epidemic in the Southeastern region with the Northeastern region.

To investigate the temporal trend in the increase of transmission clusters in Rio Grande do Sul, Paraná, Santa Catarina and São Paulo, Spearman's correlation coefficient was calculated (Figure 3B). Additionally, phylodynamic analyzes were performed to examine the dynamics of the HIV‐1C viral population through time across these states (Figure 3C). A strong positive correlation (r > 0.80; *p* < 0.05) between the number of clustered sequences and sequence isolation data was observed for the state of São Paulo (for both 3% and 2% GD threshold), indicating a significant increase in the number of intrastate transmission clusters between 1987 and 2017 (Supporting information Figure 2). Moreover, Bayesian Skygrid plot generated from sequences isolated in the state reveals that the most substantial increase in the effective population size (Ne) occurs after 2013. These results align with previous studies such as the work conducted by Pimentel et al (2024), which reported a subtype C frequency of 10% among individuals with recent diagnosis (post 2013) in São Paulo [24], significantly higher than the 3.8% frequency observed during the 2000s [47]. This pattern reveals an established and expanding epidemic of subtype C in the state of São Paulo.

In contrast, the correlation between the number of clustered sequences and sequence isolation data for the southern states were moderate or weak and not statistically significant (*p* > 0.05, Figure 3B). The number of new HIV‐1C links are not increasing for these states over the sampling time covered in this study. However, Bayesian Skygrid plots suggest a relative increase in the HIV‐1C population over time in Santa Catarina, Rio Grande do Sul and Paraná but the broad confidence intervals prevent further conclusions (Figure 3C). These findings may be an indication that the number of new HIV‐1C transmissions has remained constant in these states, meaning it has not varied over recent years, while the virus continues to spread, leading to an increase in Ne.

The strict clustering thresholds selected in this study ensure high confidence in our analysis. However, these stringent parameters may introduce bias, as they are more likely to capture recent transmission clusters. With potentially overlooking larger clusters involving long term infections, where genetic divergence has accumulated over time [48]. Additionally, although the absence of detailed individual epidemiological data (e.g., contact tracing) prevents more granular analyzes, the robust consistency between our phylogenetic results and previous studies describing transmission networks and the role of important population centers, such as São Paulo, in the Brazilian epidemiological dynamics [11, 24, 27, 49, 50], suggests that the dissemination patterns inferred from genetic data reflect real and significant population dynamics. This phylodynamic approach, while not replacing more detailed individual analyzes, such as contact tracing, offers an essential view of large‐scale viral expansion, especially in contexts with limited access to direct epidemiological data, as is the case in the Brazilian scenario. Our results, align with previous studies, characterizing the epidemic in Rio Grande do Sul as isolated, with limited influence on the national HIV‐1C landscape. In contrast, São Paulo, emerges as a major transmission hub, facilitating the spread of subtype C and bridging the southern epidemic with other regions of Brazil. The identification of Goiás as a potential secondary hub emphasizes the importance of investigating regional dynamics in underexplored areas to fully understand HIV‐1 epidemiology in Brazil.

## Author Contributions

Conceptualization was carried out by Daniel Polita, Marta Giovanetti, Luciane Amorim Santos, and Ricardo Khouri; data curation was conducted by Daniel Polita, Laise de Moraes, and Filipe Ferreira de Almeida Rego; formal analysis was performed by Daniel Polita, Dennis Maletich Junqueira, and Gabriel Carvalho; investigation was undertaken by Luciane Amorim Santos, Filipe Ferreira de Almeida Rego, Marta Giovanetti, Laise de Moraes, and Ricardo Khouri; methodology was developed by Dennis Maletich Junqueira and Ricardo Khouri; resources were provided by Ricardo Khouri and Dennis Maletich Junqueira; the original draft was written by Daniel Polita; and writing—review and editing—was completed by Laise de Moraes, Marta Giovanetti, Filipe Ferreira de Almeida Rego, Luciane Amorim Santos, Dennis Maletich Junqueira, and Ricardo Khouri. All authors have read and approved the final version of the article.

## Conflicts of Interest

The authors declare that the research was conducted in the absence of any commercial or financial relationships that could be construed as a potential conflict of interest.

## Supporting information


**Supplementary Figure** 1. Scatter plot representing the number of transmission clusters identified in HIV‐1 subtype C pol sequences phylogenetic reconstruction when applying different threshold values of genetic distance (0.005, 0.01, 0.015, 0.02, 0.025, 0.03, 0.035, 0.04, 0.045, 0.05, 0.055, 0.06) and approximate likelihood‐ratio (aLRT) SH‐like test (SH‐aLRT, 80, 85, 90, and 95). Node support values are colored according to the legend in the top‐right corner. (A) Number of clusters formed exclusively by sequences sampled in Brazil. (B) Number of sequences from Brazil, assigned to clusters formed exclusively by sequences sampled in Brazil.

**Supplementary Figure** 2. Correlation between the number of sequences within transmission clusters and the year of sampling for transmission clusters identified in São Paulo, Paraná Santa Catarina, and Rio Grande do Sul. (A) Genetic distance threshold of 3% (SH‐aLRT 85) (replicating Figure 3B); (B) Genetic distance threshold of 2% (SH‐aLRT 85).

**Supplementary Table 1.** Total number of Brazilian HIV‐1 subtype C sequences (clustered and non‐clustered) included in this study, by Brazilian state. BR NA: Brazilian sequences with no state identification.

## Data Availability

The data that support the findings of this study are available from the corresponding author upon reasonable request.
