# Supplementary material for: South‐to‐Southeast Expansion of HIV‐1 Subtype C in Brazil
Source: J Med Virol. 2025 Aug 29;97(9):e70580. doi: 10.1002/jmv.70580 (PMC12395213; doi:10.1002/jmv.70580)
Supplement: Supplementary file 3 — Supplementary Table 1. Total number of Brazilian HIV‐1 subtype C sequences (clustered and non‐clustered) included in this study, by Brazilian state. BR NA: Brazilian sequences with no state identification. [file JMV-97-e70580-s001.docx]

| **State** | **Total Sequences** | **Clustered Sequences** | **Non-Clustered Sequences** |
| --- | --- | --- | --- |
| Amapá | 5 | 3 | 2 |
| Amazonas | 15 | 3 | 12 |
| Bahia | 55 | 10 | 45 |
| Ceará | 19 | 1 | 18 |
| Distrito Federal | 3 | 1 | 2 |
| Espirito Santo | 31 | 3 | 28 |
| Goiás | 59 | 17 | 42 |
| Maranhão | 2 | 2 | 0 |
| Mato grosso do Sul | 49 | 12 | 37 |
| Minas Gerais | 53 | 14 | 39 |
| Pará | 18 | 4 | 14 |
| Paraná | 345 | 55 | 290 |
| Pernambuco | 12 | 2 | 10 |
| Rio de Janeiro | 94 | 20 | 74 |
| Rio Grande do Sul | 844 | 155 | 689 |
| Rondônia | 11 | 2 | 9 |
| Santa Catarina | 691 | 97 | 594 |
| São Paulo | 348 | 119 | 229 |
| Not Available (NA) | 68 | 25 | 43 |
| **Total** | **2722** | **545** | **2177** |
